# Recent advanced in Surface Guided Radiation Therapy

**DOI:** 10.1186/s13014-020-01629-w

**Published:** 2020-07-31

**Authors:** P. Freislederer, M. Kügele, M. Öllers, A. Swinnen, T.-O. Sauer, C. Bert, D. Giantsoudi, S. Corradini, V. Batista

**Affiliations:** 1 Department of Radiation Oncology, University Hospital, LMU Munich, Munich, Germany; 2 Department of Hematology, Oncology and Radiation Physics, Skåne University Hospital, Lund, Sweden; 3 Medical Radiation Physics, Department of Clinical Sciences, Lund University, Lund, Sweden; 4 Maastricht Radiation Oncology (MAASTRO), Maastricht, the Netherlands; 5 Department of Radiation Oncology, Universitätsklinikum Erlangen, Friedrich-Alexander-Universität Erlangen-Nürnberg (FAU), Erlangen, Germany; 6 Department of Radiation Oncology, Massachusetts General Hospital and Harvard Medical School, Boston, USA; 7 Department of Radiation Oncology, Heidelberg University Hospital, Heidelberg, Germany; 8 Heidelberg Institute of Radiation Oncology (HIRO), Heidelberg, Germany; 9 National Center for Tumor diseases (NCT), Heidelberg, Germany

**Keywords:** Surface guided radiation therapy, SGRT, Patient positioning, Motion management, Deep-inspiration breath-hold, Intra-fractional motion mitigation, Patient safety

## Abstract

The growing acceptance and recognition of Surface Guided Radiation Therapy (SGRT) as a promising imaging technique has supported its recent spread in a large number of radiation oncology facilities. Although this technology is not new, many aspects of it have only recently been exploited. This review focuses on the latest SGRT developments, both in the field of general clinical applications and special techniques.

SGRT has a wide range of applications, including patient positioning with real-time feedback, patient monitoring throughout the treatment fraction, and motion management (as beam-gating in free-breathing or deep-inspiration breath-hold). Special radiotherapy modalities such as accelerated partial breast irradiation, particle radiotherapy, and pediatrics are the most recent SGRT developments.

The fact that SGRT is nowadays used at various body sites has resulted in the need to adapt SGRT workflows to each body site. Current SGRT applications range from traditional breast irradiation, to thoracic, abdominal, or pelvic tumor sites, and include intracranial localizations.

Following the latest SGRT applications and their specifications/requirements, a stricter quality assurance program needs to be ensured. Recent publications highlight the need to adapt quality assurance to the radiotherapy equipment type, SGRT technology, anatomic treatment sites, and clinical workflows, which results in a complex and extensive set of tests.

Moreover, this review gives an outlook on the leading research trends. In particular, the potential to use deformable surfaces as motion surrogates, to use SGRT to detect anatomical variations along the treatment course, and to help in the establishment of personalized patient treatment (optimized margins and motion management strategies) are increasingly important research topics. SGRT is also emerging in the field of patient safety and integrates measures to reduce common radiotherapeutic risk events (e.g. facial and treatment accessories recognition).

This review covers the latest clinical practices of SGRT and provides an outlook on potential applications of this imaging technique. It is intended to provide guidance for new users during the implementation, while triggering experienced users to further explore SGRT applications.

## Introduction

In recent years, the clinical use of Surface Guided Radiation Therapy (SGRT) using optical surface scanning for patient positioning, intra-fraction motion monitoring and respiratory gating techniques has increased. In general, SGRT systems use a combination of a projector and one or several camera units to register a real-time 3D surface of the patients. A reference surface relative to the treatment isocenter position is used to calculate the necessary correction of the patient position in translational and rotational directions. There are four main optical surface scanning technologies used in radiotherapy, laser scanners [1], time-of-flight systems [2, 3], stereovision systems [4] and structured light systems [5, 6]. Optical surface scanners, with their high spatial and temporal resolution, have proven to be an important addition to the radiation therapy process regarding patient positioning and monitoring [1, 7, 8].

SGRT can be seen as a “four-eyes principle” tool that allows continuous monitoring of patient positioning, thus improving patient safety [9, 10] and comfort [11, 12], and at the same time standardizing workflows (higher precision and reproducibility) [13]. Additionally, it has the potential to improve the clinical outcomes through accurate target irradiation [14], while sparing normal tissue [15].

In terms of patient positioning, SGRT is an effective tool for the reduction of the overall treatment time, and minimizing of imaging dose, as: i) it provides in-room online information of the complete surface and position of the patient, ii) for superficial tumors (where surface deviations can act as a surrogate for tumor motion) SGRT allows for a more accurate positioning compared to 3-point-lasers and might allow to reduce the number of daily imaging in some cases [16]; iii) for deeper located tumors (with no direct correlation between surface deviations and tumor movement) daily imaging remains mandatory, but SGRT can reduce the time required for image registration and prevent the need for multiple imaging [17]. Although imaging dose could be seen as negligible compared to the whole body scatter dose from a photon treatment, SGRT can be identified as one image-guided step that can be accomplished without ionizing radiation as proposed in AAPM TG 75 for imaging dose reduction [18].

As SGRT systems provide real-time motion monitoring of the patient surface throughout the whole treatment fraction, an additional level of safety is added to radiotherapy workflows. The beam can be held if parts of the patient’s surface deviate from the reference position based on the planning CT set-up or if the calculated isocentric deviations exceed a certain threshold.

One of the most promising applications of SGRT is gated radiotherapy delivery of tumor locations close to the skin surface (e.g. breast cancer) by means of Deep-Inspiration-Breath-hold (DIBH) and voluntary-DIBH (vDIBH). Another application developed in recent years is the use of SGRT in whole-brain radiation therapy (WBRT) or stereotactic radiosurgery (SRS). Here, an SGRT system has the ability to monitor the surface of the patient within an open-face immobilization mask in planar and non-coplanar treatments [19]. SGRT has also been applied in special techniques such as accelerated partial breast irradiation (APBI), stereotactic body radiation therapy (SBRT), and with limited use in pediatrics [20].

The present review aims to provide a summary of recent clinical advances in SGRT and the latest research findings applied to modern SGRT-based treatments, with an outlook on future application fields.

### Clinical applications

**Fig. 1:**
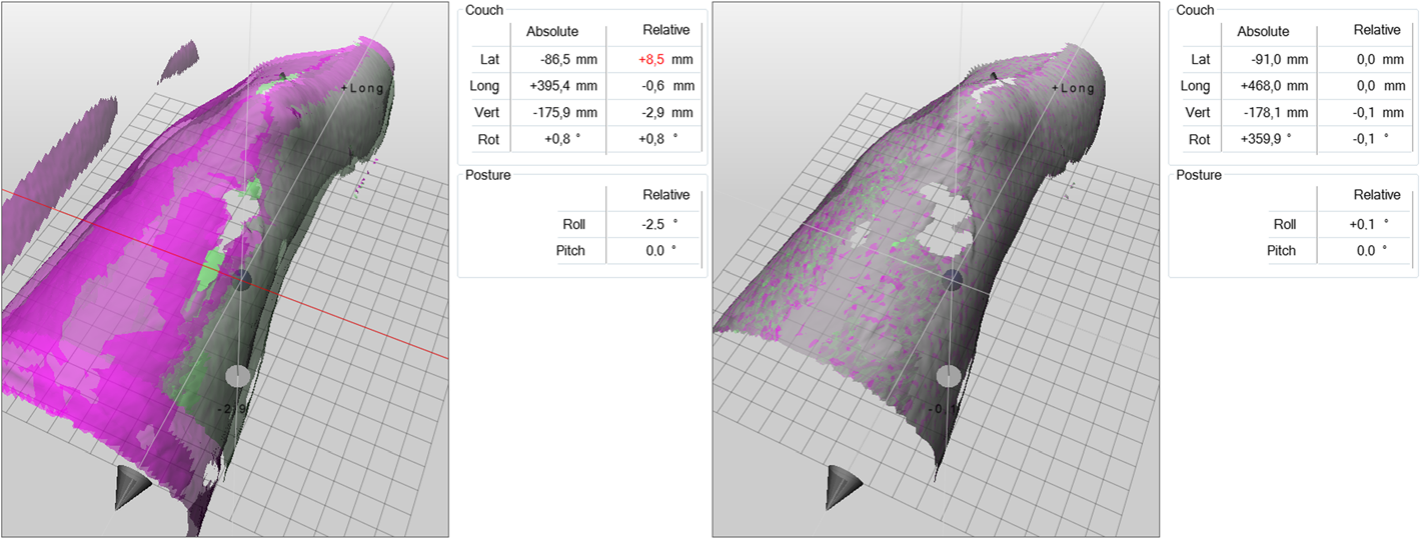
Example of patient positioning using SGRT. Left: The live surface data from a whole left leg (purple) deviates from the reference surface (green). Right: After correction, the surface data matches the reference image. Image courtesy of LMU University Hospital Munich, Germany

SGRT has become a common tool in radiotherapy due to its ability to provide real-time, 6-dimensional patient positioning and monitoring, as exemplarily illustrated in Fig. 1. Daily imaging of the patient without concern for radiation dose and the potential for reduced setup times are the main advantages of the technology. In order to identify the clinical benefits of surface imaging for patient setup, several investigations have been conducted which benchmark the patient setup accuracy using verification imaging. There is a broad agreement on the supremacy of SGRT over 3-point laser-based patient setup, when the surface serves as a surrogate for the target positioning, i.e. breast and other skin-near targets [13, 16, 21–30]. For deeper situated targets, surface imaging should be used with caution [13, 22, 31, 32].

#### Breast positioning

Regarding setup of breast cancer patients, studies comparing laser alignment with surface imaging report a reduction of positioning errors for skin and clip alignment [23–26] by around 40% on average, with absolute errors (1SD) for all studies being smaller than 4 mm, verified with kV orthogonal imaging [29, 30, 33, 34], portal imaging [16, 27, 30], or cone-beam computed tomography (CBCT) [13]. In addition to accurate isocenter positioning, the surface imaging provides guidance for correcting patient posture, i.e. chin and arm position. In this context, it has been shown that surface-guided correction of the arm posture also improved the breast position [13, 16, 27, 30]. Nevertheless, some factors can affect the accuracy in surface-guided patient setup, such as patient motion [35], surface shadowing [16], selection of the region-of interest (ROI) [14], absence of anatomical gradients in the patient (e.g. very flat surfaces) and anatomical changes throughout the treatment [21, 28, 36]. The SGRT workflow within an NAL (No Action Level) imaging protocol has shown the potential to reduce the frequency of IGRT, and thus spare additional imaging dose [16, 30, 31]. Advances in surface imaging for positioning of breast cancer patients have recently led to a tattoo-free workflow and reduced time for patient setup compared to laser alignment [26].

#### Positioning of internal targets (abdomen/pelvis/Head& Neck)

Several studies have shown a poor correlation between movement of the patient surface and movement of internal targets [13, 22, 37, 38]. Large shifts up to about 3 cm were observed for targets in the abdomen, and up to about 2 cm for pelvis and lower extremities [13, 22, 31]. However, surface imaging achieved at least the same precision as laser alignment and was considered a valuable tool for initial patient setup and as a complement to conventional imaging modalities [13, 22, 38]. The lack of correlation between the surface and internal structure movement is not an argument against the use of SGRT [32], since SGRT does not provide contradictory but complementary information for image guidance. The choice of the ROI for the reference image influences the correlation between SGRT and image-guided positioning. In pelvic SGRT, improved agreement with CBCT was found by excluding deformable anatomies such as the stomach from the reference image ([39]). In contrast, only minor displacements were observed in the head and neck region [22, 31]. Haraldsson et al. reported significantly reduced imaging times for patient positioning using surface imaging in head and neck patients by 5 min per fraction [31].

#### Intracranial tumors: WBRT & SRS

Traditionally, WBRT has been the recommended treatment option for patients with more than three intracranial brain metastases (BM), but WBRT can cause long-term adverse events (e.g. neurocognitive decline) and a reduced quality of life [40]. In contrast, for a limited number of metastases [1–4], stereotactic radiosurgery is the option of first choice in most cases. Recent technological advances have made linac-based frameless SRS a more patient friendly treatment option, allowing for accurate patient positioning and shorter treatment times [41–45]. Since SRS is increasingly used in patients with multiple BM, it is important that SRS is performed with the highest quality achievable to avoid complications such as radionecrosis. Therefore, it is essential to treat the BM with smaller treatment margins, which requires a 6DOF correction under the guidance of on-board CBCT and a thermoplastic mask [46]. This improved patient setup makes it possible to treat multiple lesions with a single isocenter, which in combination with flattening filter free (FFF) beams can further decrease the beam-on time [47, 48].

**Fig. 2:**
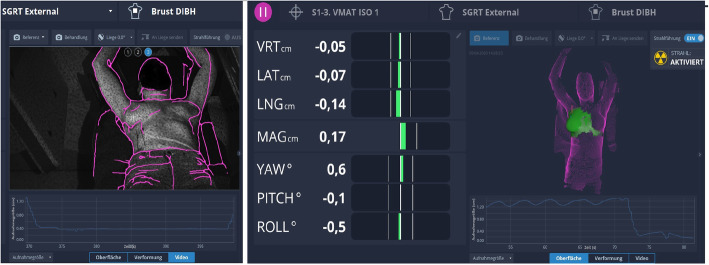
**a** ORFIT open face mask together with a T-shaped vacuum bag, **b** Catalyst HDTM in kV-MV setup using the ExaFix-3 baseplate and **c** in setup at couch 0°(3 cameras are indicated with arrows). The cropped surface image (**d**) is extracted from the patient’s open face mask (**e**). Image courtesy of MAASTRO Clinic, Maastricht, The Netherlands

The advantages of including non-coplanar couch angles in the treatment planning can result in better sparing of normal brain tissue. This approach has already been extensively published [47, 49]. However, with modern Linacs it is not possible to verify the patient’s position using CBCT at non-coplanar couch angles (Fig. 2). Additional equipment such as the ExacTrac® X-ray system (Brainlab AG, Munich, Germany) is able to provide accurate intrafraction setup information of the bony anatomy of the patient – regardless of the couch angle, but the imaging procedure adds some additional dose and extends the overall treatment time [50, 51]. Tarnavski et al. showed that although patients were immobilized with thermoplastic masks, positioning corrections exceeding 1 mm appeared in 42% of the beams and exceeding 1 degree in 9% of the beams [52].

A clear advantage of SGRT systems is the lack of radiation dose and the procedure only minimally increases the overall treatment time [8]. Optimal use of SGRT systems in patients with brain tumors requires the use of open-face masks or even no masks. Dekker et al. described the results of 30 palliative patients treated with WBRT without any immobilization mask [53]. With a success rate of 93%, the authors concluded that radiation therapy without a thermoplastic mask was clinically feasible. Under guidance of a single camera system (C-RAD, Uppsala, Sweden), the isocenter motion was within 1.1 mm on average. Obviously, the system is interconnected with the Linac and can perform an automated beam shut off if the patient moves out of a predefined threshold.

For treatments with non-coplanar couch angles, accurate knowledge about the coincidence between radiation and couch rotation isocenter becomes important, as it is not possible to correct for couch walkout during treatment delivery. Regular Winston-Lutz checks provide accurate information about the radiation isocenter for different gantry, collimator and couch angles and can also indirectly provide information about the accuracy of the system [19, 54, 55].

#### Breath-hold

Since respiratory-induced organ motion is considered the largest intrafractional organ motion, the uncertainty during radiation therapy of tumors or lesions which are affected by respiration has to be taken into account. DIBH (maximum inspiration) or a shallow BH (moderate inspiration) are effective methods of respiratory motion mitigation [56]. Both concepts reduce tumor motion to a minimum and allow for a reduction in dose to the heart with similar planning target volume (PTV) coverage [57]. For lung or liver treatments, the breath-hold level can be monitored using SGRT systems, although the system can only monitor the patient surface as a surrogate for tumor motion at most. On the other hand, respiratory motion can also be used to increase OAR sparing for specific tumor sites, in which DIBH increases the distance between the PTV and the OARs [58]. For example, in breast cancer the DIBH technique leads to a reduction in cardiac dose, especially to radiosensitive structures like the left anterior descending artery (LAD) where any additional dose increases the risk of coronary artery disease and risk of ischemic heart disease [59–63]. Laaksomaa et al. reported the possibility of portal imaging reduction for whole breast DIBH with residual errors of ≤3 mm [30]. Furthermore, DIBH reduces the interplay effect and improves plan robustness [56] and reduces the dose to LAD and heart significantly in proton therapy for breast [64] and Hodgkin’s lymphoma [65]. Left breast cancer irradiation using DIBH using optical surface scanners is nowadays widely implemented at multiple institutions [66] and the concept has also been adapted to right-sided breast irradiation for a reduction of lung and liver dose [67].

SGRT systems offer the possibility of evaluating intra-DIBH stability, which has been reported with ≤0.7 mm, together with an intra-fractional reproducibility of ≤2.2 mm [68] and with 0.3 mm as the median standard deviation of the BH level during DIBH [69]. Kügele et al. reported that the intrafractional reproducibility for tangential and locoregional treatment was as low as 1 mm (median over 40 patients) in all three translational directions, but during a single treatment session the maximum deviation was up to 5 mm, which resulted in large effects on the target coverage and OAR doses [70]. The accuracy of SGRT systems have been reported within 5 mm for DIBH positioning and monitoring [71], and are similar to those reported in studies using spirometry-based positioning [68].

**Fig. 3:**
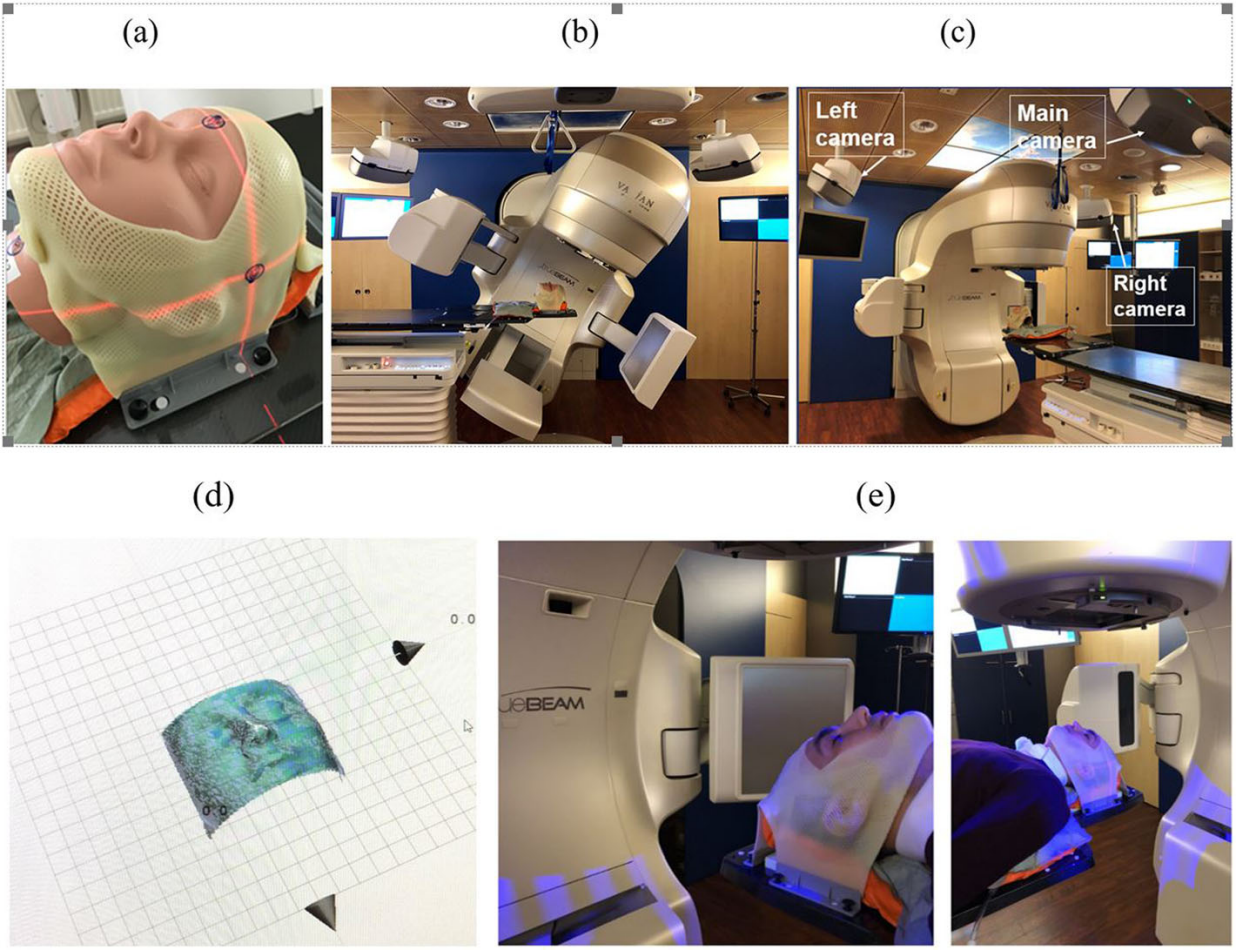
Positioning of a DIBH patient. Left: Positioning of the patient using the reference surface (purple) and the live surface data. Right: Monitoring of the DIBH during treatment on a highlighted (green) ROI. The breathing curve is depicted on the bottom. Image courtesy of Heidelberg University Hospital, Germany

**Fig. 4:**
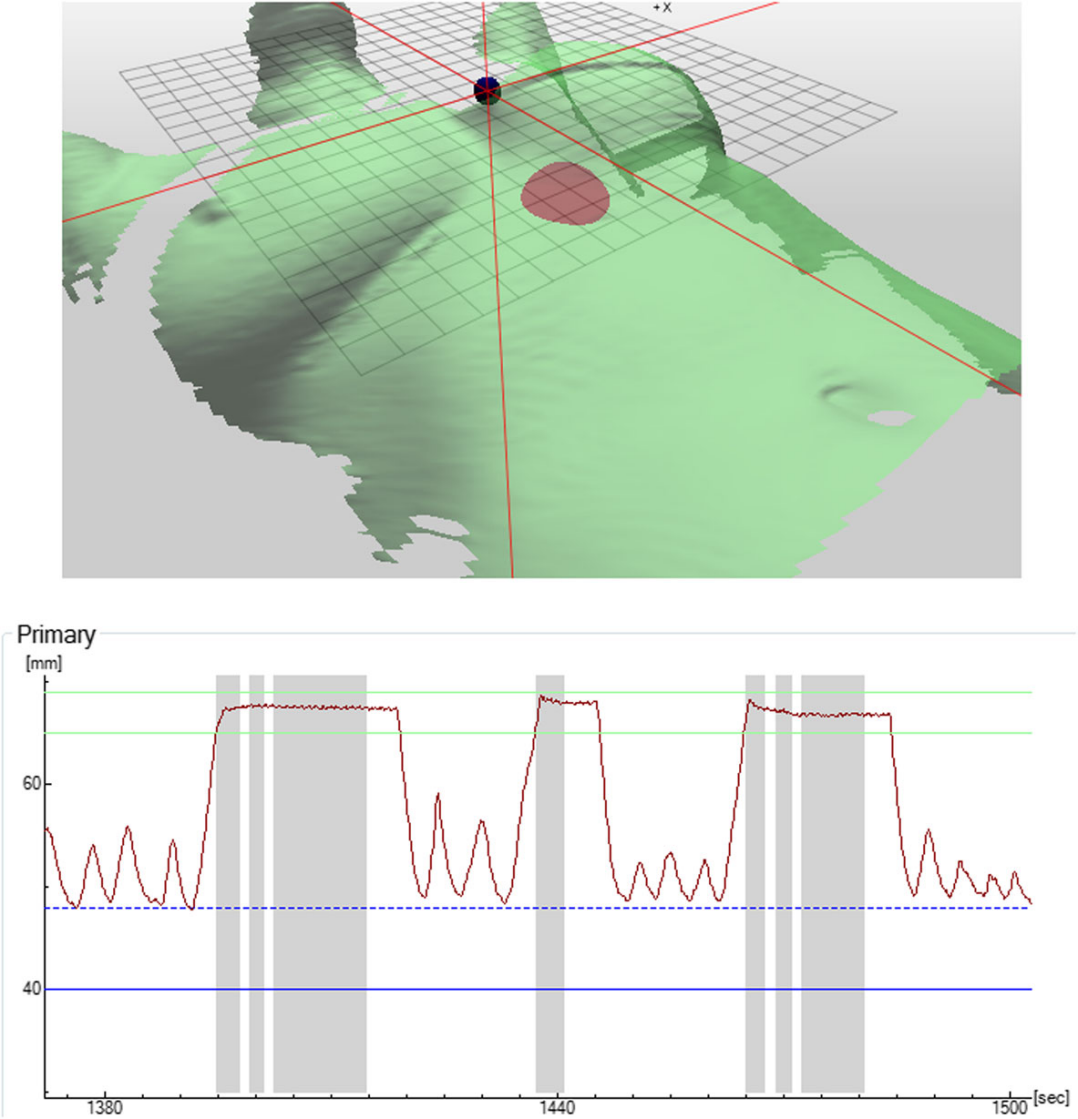
DIBH monitoring. Top: A breathing spot (red) on the patient surface (green) is monitored. Bottom: The breathing curve with three breath-holds, covering 7 individual beams (indicated using the grey bars). Image courtesy of LMU University Hospital Munich, Germany

A reduction in treatment time using SGRT for DIBH has not been consensually reported, but an improved safety aspect as patient positioning, intra-DIBH stability, and intrafractional DIBH reproducibility can be measured directly and accounted for using an adaption of setup margins and beam control. Figures 3 and 4 show examples of DIBH patient positioning and monitoring.

### Special techniques

Accelerated partial breast irradiation (APBI).

In APBI the treatment target is limited to the post-surgical cavity of patients with early-stage breast cancer where surgical clips can be implanted for target localization via x-ray imaging. SGRT has been shown to improve setup for this soft tissue target [72] and a strong correlation of the external patient surface with the internal resection site has been suggested [24]. In a recent study, the target registration error (TRE) for a SGRT system agreed with the kV clip-based TRE within less than 3 mm. Furthermore, the combination of SGRT and 2D X-ray matching to surgical clips allowed for accurate alignment and setup verification without the need for skin-based tattoos [26]. In the absence of surgical clips in the lumpectomy area, SGRT still appears to be a potentially valuable image guidance approach for patients treated with APBI when they experience < 10% changes in surgical cavity volume between computed tomography (CT) simulation and treatment [21].

#### Motion management and SBRT

Almost one third of all clinical SGRT users have implemented respiratory gating for SBRT treatments or conventional lung or abdominal RT, for patient positioning and/or motion management [32]. SGRT can improve pre-imaging treatment setup, decrease the necessity of orthogonal kV imaging prior to CBCT [73] and is promising in detecting deviations between pre- and post-treatment setup in SBRT [74]. Four-dimensional computed tomography (4DCT) acquisition and treatment delivery under surface guidance showed excellent temporal accuracy and consistency to the Varian RPM system in motion-tracking studies [75–77] and has the potential to improve respiratory motion management [78]. Kauweloa et al. observed an increased uncertainty in mean-position tracking of surface-guided 4DCT-acquisition with decreasing amplitude of the breathing pattern, suggesting that the SGRT system might be more appropriate for phase- rather than amplitude-sorting of 4DCT [76]. The potential of SGRT for respiratory motion monitoring and motion management is demonstrated by the strong correlation of SGRT monitoring with internal tumor position monitoring by x-ray imaging [79, 80]. Respiratory motion signal and estimated volume variations are well correlated with spirometer measurements [81–83]. However, when implementing a SGRT-based tumor-tracking or gating-system, careful characterization of the beam-on and beam-off delays is advisable, as these might be non-negligible and vary between the SGRT and beam delivery systems [84–86].

#### Pediatric

SGRT-based intra-fraction monitoring of pediatric treatments is not widely used [32] and literature is limited. In a case report [87] using SGRT in combination with a linac using the FFF mode, the palliative radiation treatment of an 18-month old boy with relapsed Wilms tumor was reported. He had a large anterior mediastinal mass which critically obstructed his airway. SGRT treatment could be delivered in sufficiently short time without anesthesia. SGRT systems have been added as a safety feature in pediatric treatments to assist in patient setup and provide additional error detection [88].

#### Charged particles

In proton therapy breast treatments, SGRT combined with initial and weekly in-beam X-ray imaging, has been proven to be a safe replacement of daily orthogonal X-ray images for patient positioning, resulting in shorter setup time, and reduction of imaging dose for the patient [14, 58, 89]. With this approach, significant discrepancies (> 3 mm shifts) between surface and radiographic imaging indicate changes in breast anatomy. A further application in proton therapy is the verification of the nozzle setup [90], by air gap and source to surface distance (SSD) calculations, which provide a way to confirm the physical target depth at treatment.

### Quality assurance (QA) – update on current status

SGRT systems require a demanding Quality Assurance (QA) program in order to achieve submillimeter accuracy and reliable functioning. Not only the system performance itself, but also the associated workflow needs to be ensured. AAPM TG 147 [91] set the first guidelines about this topic. However, after 8 years of technological development and a wider usage of SGRT by an increased number of facilities, there is a need for updated QA guidelines.

Applications of hypofractionated RT (e.g. SRS, SBRT) require measurements of the static accuracy with 6DOF [92], differentiation between single or multiple isocenter plans [93], including couch rotations, walkout effects, and the impact of miscalibration [19, 94].

Additionally, the gain in the frame-rate of the newest SGRT systems attracts the use to treat patients under free-breathing, where parameters as gating response times, gating window and Linac configurations (e.g. dose rate, beam hold times) need to be included into the QA program [86, 95, 96]. Even more complex, is the application of this technology for particle therapy, specifically in pencil beam delivery, where parameters as latency, the impact of room geometries into the system performance and potential prediction algorithms, when using beam-gating or -tracking [97, 98] need to be taken into account. Also in ring-like Linacs, a QA program needs to be implemented [37, 99, 100], as well as when the surface is used as a surrogate for 4D-acquisitions [101–104].

Although the majority of the users use commercially available phantoms, some features are currently impossible (or challenging) to test, such as deformable registration algorithms [7, 36], latency time [105], skin colors and room lightening, full-workflow integration [106], and thermal signature.

### Future applications

The number of SGRT users and applications has increased rapidly in recent years and is expected to further increase in the coming years. Considering the discomfort and emotional burden of tattoos, which could be a constant reminder of a patient’s cancer treatment, a complete replacement of patient’s tattooing with a markerless SGRT-based workflow could be a real prospect in the near future.

Deformable surface registration, in combination with extensive studies on the correlation of patient surface motion to internal organ and tumor motion, can constitute a more accurate method for recording and monitoring patient motion and anatomy changes. Motion monitoring for DIBH, gated or even treatments with tumor tracking, can benefit from the improved data that SGRT offers in comparison to 1D surrogates. Surface data acquired during CT simulation can help to stratify patients to motion mitigation techniques, for example by quantifying the patient’s ability to follow the instructions required for a certain breath hold technique. Also, with respect to inter-fractional anatomical changes, such as weight loss or lymphedema in breast treatments, SGRT offers a potential. With the upcoming possibility of thermal tracking (ExacTrac Dynamic, Brainlab AG, Germany), even the judgement of physiologic processes, such as inflammation or early skin toxicity might be accessible.

Along with the retrospective analysis of patient motion, resulting from intra-fractional monitoring data, the SGRT systems provide the information of the setup variability, which combined with anatomical imaging can determine the necessary information to establish site- or even patient-specific treatment margins. It is therefore expected that SGRT will increasingly contribute to the development of personalized patient care and adaptive radiotherapy treatments, with the potential to decrease the volume of irradiated healthy tissues and the total number of verification imaging (X-ray or CBCT) required to control inter-fractional variations. SGRT can either replace the kV imaging itself or trigger kV images when changes over a certain threshold are detected.

**Fig. 5:**
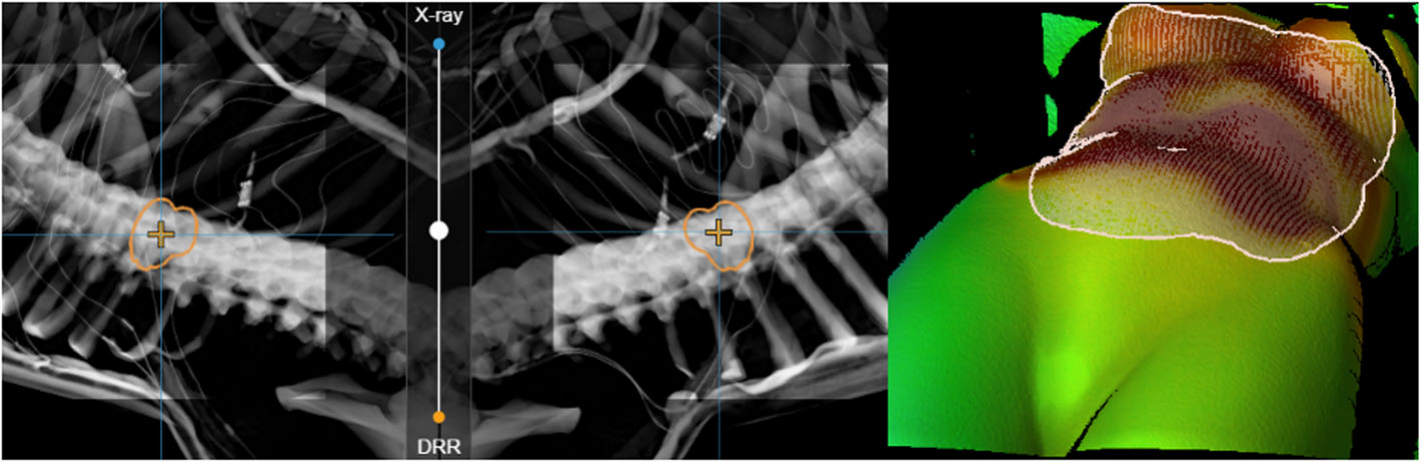
A combination of X-ray monitoring (left) and SGRT guidance (right). A phantom with a distinct heat signature has been used for demonstration purposes. SGRT and X-ray tracking are combined in a single workflow. Image courtesy of LMU University Hospital Munich, Germany

Recently, a combination of SGRT with X-ray monitoring in a dedicated system has been introduced (ExacTrac Dynamic, Brainlab AG, Germany). The combined workflow could enables SGRT-guided patient positioning and intrafractional motion monitoring with the possibility of positioning the patient also according to the internal anatomy. An automatic triggering of X-ray images allows to verify the internal position of the target, when the patient surface exceeds a certain tolerance. As an addition to the surface information, a thermal signature of the surface is used to increase registration accuracy, as shown exemplarily in Fig. 5.

The determinant role of SGRT in terms of adaptive planning can be of greater importance for particle therapy, where undetected patient anatomy and posture changes, such as inflammation, weight increase or loss, may have more substantial dosimetric effects due to the strong influence of changes in particle range [107, 108]. However, SGRT application in particle therapy can be expected to further extend also in machine and patient-specific QA. Due to the extensive weight of the gantry configuration, the mechanical and radiation isocentricity in most proton therapy systems strongly depends on complicated algorithms to adjust the couch position as a compensation for gantry sagging, which varies according per treatment angle. A well-calibrated surface imaging system with a system-specific QA routine could be implemented as a secondary verification procedure for couch-positioning verification, but also as an independent check of gantry and couch position (and motion in case of VMAT) for photon beam therapy.

Furthermore, SGRT systems could be integrated into the entire clinical radiation therapy workflow, considering the recent advances in biometrics and face recognition algorithms. SGRT is able to provide patient registration upon arrival via facial recognition, daily verification of treatment accessory items at the linac and verify their correct position on the couch, moreover it can facilitate patient-specific and machine QA.

For each of the enumerated applications, a dedicated QA program, including the SGRT-system and the peripherals (i.e. TPS, Linac, imaging systems) must be implemented. Development of suitable phantoms, integration in an analysis framework and parameterization of the system quality are some of the fields to be explored.

## Conclusion

The use of SGRT has shown to provide increased patient safety throughout the course of the radiotherapy treatment and increased accuracy for the treatment of specific anatomic sites. SGRT can be considered an additional safety tool, for example for intra-fractional motion management, but certain techniques such as DIBH or open masks treatments have made extraordinary progress through the use of SGRT.

## Data Availability

Not applicable.

## Abbreviations

4DCT: Four-dimensional computed tomography
APBI: Accelerated partial breast irradiation
BM: Brain metastases
DIBH: Deep-Inspiration-Breath-hold
CBCT: Cone-beam computed tomography
CT: Computed tomography?
FFF: Flattening filter free
NAL: No Action Level
QA: Quality Assurance
ROI: Region-of interest
PTV: Planning target volume
SBRT: Stereotactic body radiation therapy
SGRT: Surface Guided Radiation Therapy
SRS: Stereotactic radiosurgery
SSD: Source to surface distance
TRE: Target registration error
vDIBH: Voluntary-DIBH
WBRT: Whole-brain radiation therapy
