# Correction to: Recent advances in Surface Guided Radiation Therapy

**DOI:** 10.1186/s13014-020-01661-w

**Published:** 2020-10-24

**Authors:** P. Freislederer, M. Kügele, M. Öllers, A. Swinnen, T.-O. Sauer, C. Bert, D. Giantsoudi, S. Corradini, V. Batista

**Affiliations:** 1 Department of Radiation Oncology, University Hospital, LMU Munich, Munich, Germany; 2 Department of Hematology, Oncology and Radiation Physics, Skåne University Hospital, Lund, Sweden; 3 Medical Radiation Physics, Department of Clinical Sciences, Lund University, Lund, Sweden; 4 Maastricht Radiation Oncology (MAASTRO), Maastricht, the Netherlands; 5 Department of Radiation Oncology, Universitätsklinikum Erlangen, Friedrich-Alexander-Universität Erlangen-Nürnberg (FAU), Erlangen, Germany; 6 Department of Radiation Oncology, Massachusetts General Hospital and Harvard Medical School, Boston, USA; 7 Department of Radiation Oncology, Heidelberg University Hospital, Heidelberg, Germany; 8 Heidelberg Institute of Radiation Oncology (HIRO), Heidelberg, Germany; 9 National Center for Tumor diseases (NCT), Heidelberg, Germany

**Correction to: Radiat Oncol 15, 187 (2020)**

**https://doi.org/10.1186/s13014-020-01629-w**

Following publication of the original article [1], the authors identified an error in the order of Figs. 2 and 3. The correct representation of figures is provided below.

The original article [1] has been corrected.

**Fig. 2:**
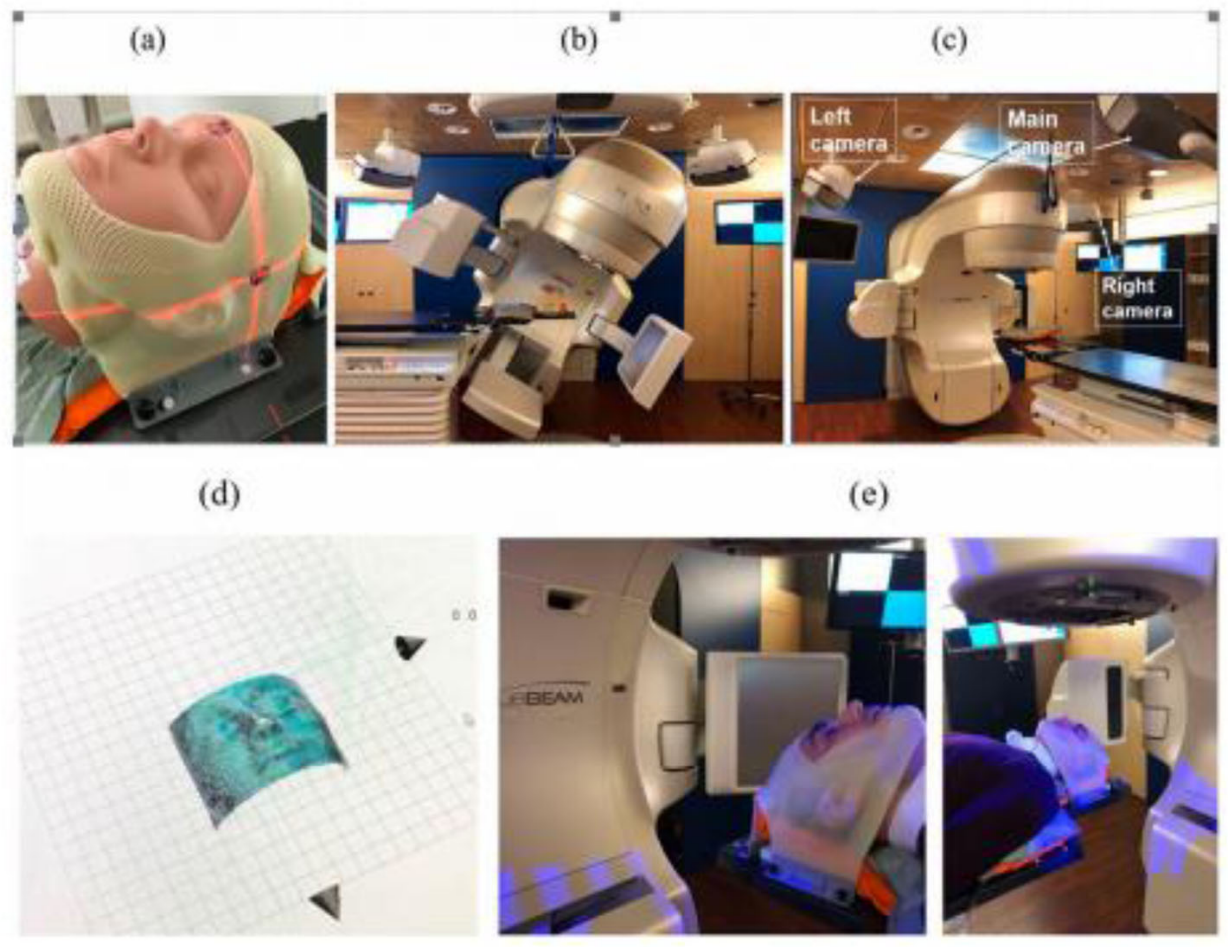
**a** ORFIT open face mask together with a T-shaped vacuum bag, **b** Catalyst HDTM in kV-MV setup using the ExaFix-3 baseplate and **c** in setup at couch 0°(3 cameras are indicated with arrows). The cropped surface image (**d**) is extracted from the patient’s open face mask (**e**). Image courtesy of MAASTRO Clinic, Maastricht, The Netherlands

**Fig. 3:**
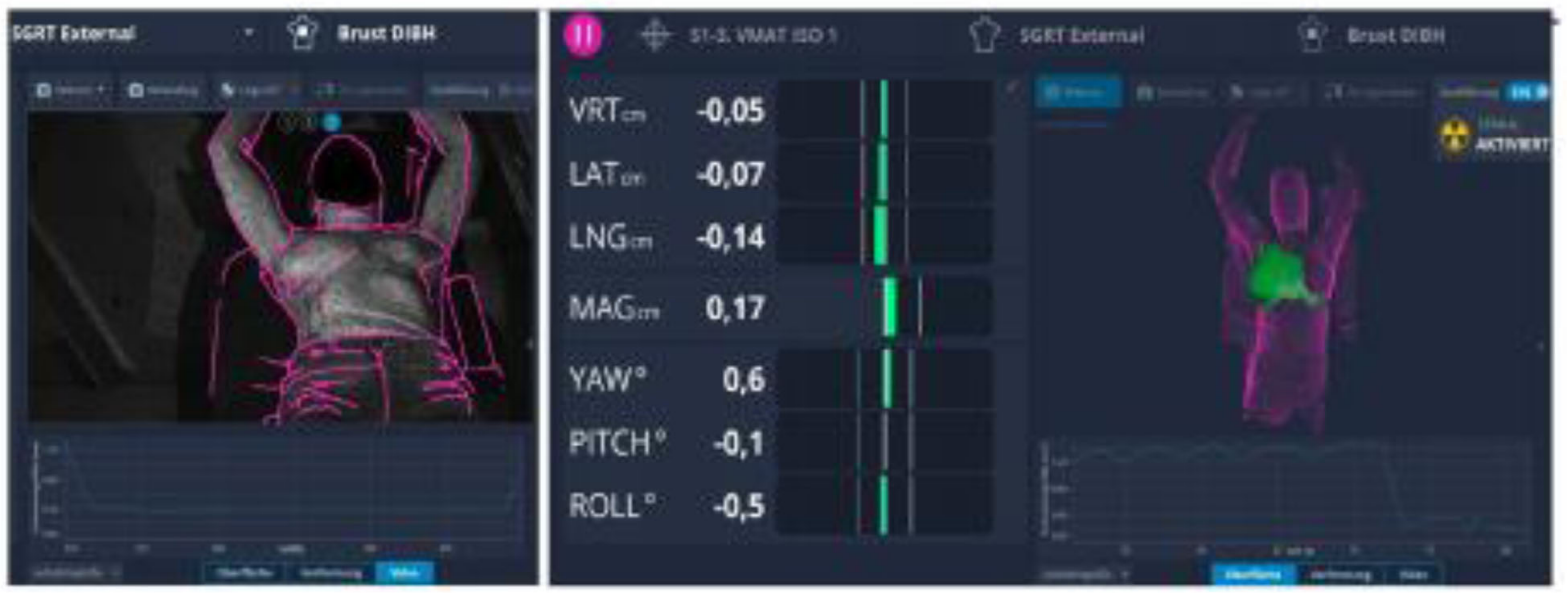
Positioning of a DIBH patient. Left: Positioning of the patient using the reference surface (purple) and the live surface data. Right: Monitoring of the DIBH during treatment on a highlighted (green) ROI. The breathing curve is depicted on the bottom. Image courtesy of Heidelberg University Hospital, Germany
